# Lenalidomide and Programmed Death-1 Blockade Synergistically Enhances the Effects of Dendritic Cell Vaccination in a Model of Murine Myeloma

**DOI:** 10.3389/fimmu.2018.01370

**Published:** 2018-06-18

**Authors:** Manh-Cuong Vo, Sung-Hoon Jung, Tan-Huy Chu, Hyun-Ju Lee, Thangaraj Jaya Lakshmi, Hye-Seong Park, Hyeoung-Joon Kim, Joon Haeng Rhee, Je-Jung Lee

**Affiliations:** ^1^Research Center for Cancer Immunotherapy, Chonnam National University Hwasun Hospital, Hwasun, South Korea; ^2^Department of Hematology-Oncology, Chonnam National University Hwasun Hospital and Chonnam National University Medical School, Hwasun, South Korea; ^3^Research Institute, VaxCell-Bio Therapeutics, Hwasun, South Korea; ^4^Department of Microbiology and Clinical Vaccine R&D Center, Chonnam National University Medical School, Hwasun, South Korea

**Keywords:** myeloma, dendritic cells, lenaldiomide, anti-PD-1, cancer immunotherapy

## Abstract

The therapeutic efficacy of dendritic cell (DC)-based immunotherapy may be potentiated in combination with other anticancer therapies that enhance DC function by modulating immune responses and the tumor microenvironment. In this study, we investigated the efficacy of DC vaccination in combination with lenalidomide and programmed death (PD)-1 blockade in a model of murine myeloma. MOPC-315 cell lines were injected subcutaneously to establish myeloma-bearing mice and the following five test groups were established: PBS control, DCs, DCs + lenalidomide, DCs + PD-1 blockade, and DCs + lenalidomide + PD-1 blockade. The combination of DCs plus lenalidomide and PD-1 blockade more potently inhibited tumor growth compared to the other groups. This effect was associated with a reduction in immune suppressor cells (such as myeloid-derived suppressor cells, M2 macrophages, and regulatory T cells) and an increase in immune effector cells [such as CD4^+^ and CD8^+^ T cells, natural killer (NK) cells, and M1 macrophages] in the spleen. Functional activities of cytotoxic T lymphocytes and NK cells were also enhanced by the triple combination. Levels of immunosuppressive cytokines, such as TGF-β and IL-10, were significantly reduced in the tumor microenvironment. These findings suggest that the combination of DCs plus lenalidomide and PD-1 blockade synergistically establishes a robust anti-myeloma immunity through a two-way mechanism, which inhibits immunosuppressive cells while activating effector cells with superior polarization of the Th1/Th2 balance in favor of the tumor immune response. This result should provide an experimental ground for incorporating check point inhibitors to existing immunotherapeutic modalities against multiple myeloma.

## One-Sentence Summary

A combination of antigen-loaded dendritic cell (DC) vaccination plus lenalidomide and programmed death (PD)-1 blockade synergistically enhanced anticancer immunity in a model of murine multiple myeloma by inhibiting immunosuppressive cells and stimulating effector cells.

## Introduction

Multiple myeloma (MM) is characterized by the infiltration of clonal malignant plasma cells in the bone marrow (BM) (1, 2). Despite advances in treating MM using novel therapies and hematopoietic stem cell transplantation, most patients experience relapses caused by immune evasion among the tumor, immune system, and tumor microenvironment (3). Thus, new therapeutic options with the potential to overcome impaired immune surveillance are needed.

Dendritic cells (DCs) are the most potent antigen-presenting cells (APCs) and play a key role in inducing and maintaining antitumor immunity. DCs are able to recognize, process, and present tumor antigens to generate antigen-specific cytotoxic T lymphocytes (CTLs) (4–9). Immune cells in myeloma patients have quantitative and functional deficiencies that contribute to myeloma-associated immune tolerance (10, 11). By contrast, the function of DCs from patients with MM can be recovered and enhanced by *ex vivo* culture (12–14). Lenalidomide is an immunomodulatory agent that targets tumor cells under immunosuppressive microenvironment (15–20). Our previous studies demonstrated that the combination of DC vaccination and lenalidomide synergistically enhanced antitumor immune responses in mouse tumor models (21, 22). Programmed cell death-1 (PD-1, CD279) and its ligands [either PD-L1 (B7-H1, CD274) or PD-L2 (B7-DC, CD273)] play a fundamental role in tumor immune escape by inhibiting effector functions (23–26). The PD-1/PD-L1 blockade was recently found to effectively treat cancer by improving durable response rates and the survival profile with minimal toxicity, suggesting that blockade can be used as a cancer therapeutic agent (27–31). However, recent studies reported that PD-1 blockade alone is insufficient to stimulate anti-myeloma immunity in clinical treatment (32, 33). Thus, combination approaches with immune-checkpoint blockade and therapies that stimulate myeloma-reactive T cells can be effective tools to treat myeloma. Such as with immunomodulatory drugs, cellular therapies are currently being applied in clinical trials. Previous studies demonstrated that lenalidomide reduces the expression of PD-1 on natural killer (NK) cells, helper cells, and CTLs, and inhibits PD-L1 expression on tumor cells and myeloid-derived suppressor cells (MDSCs) in patients with MM (20, 34). Moreover, the combination of lenalidomide and PD-1 or PDL-1 blockade increased IFN-γ expression by BM-derived effector cells in myeloma and were associated with increased apoptosis of MM cells (35).

Thus, in this study, we investigated whether the combination of DCs plus lenalidomide and PD-1 blockade has a synergistic effect in a murine myeloma model. The results demonstrate that this combination enhanced antitumor immunity by inhibiting immunosuppressive cells and cytokines as well as activating and recovering effector cells with superior polarization toward Th1 immune response. This study provides a framework for developing a more advanced immunotherapeutic modality employing DCs, lenalidomide, and PD-1 blockade to inhibit tumor cell growth as well as restore immune functionin MM.

## Materials and Methods

### Mice and Tumor Cell Lines

6- to 8-week-old female BALB/c (H-2^d^) mice were purchased from Orient Bio (Iksan, Republic of Korea) and maintained under specific pathogen-free conditions. All animal care, experiments, and euthanasia protocols were approved by the Chonnam National University Animal Research Committee. The murine MOPC-315 plasmacytoma cell line and the YAC-1 cell line were purchased from the American Type Culture Collection (Rockville, MD, USA). Cell lines were maintained in Dulbecco’s Modified Eagle’s Medium (Gibco-BRL, Grand Island, NY, USA) supplemented with 10% (v/v) fetal bovine serum (FBS; Gibco-BRL) and 1% (w/v) penicillin/streptomycin (PS).

### Immunomodulatory Drug (Lenalidomide) and Programmed Death-1 (Anti-PD-1)

Lenalidomide (Revlimid^®^) was donated by Celgene Corporation (Summit, NJ, USA) and dissolved in dimethyl sulfoxide (DMSO) to 100 mg/mL immediately before use. For injection into mice, lenalidomide stock solutions were diluted in sterile 0.9% (v/v) normal saline to a final concentration of 10 mg/mL. The final concentration of DMSO in all experiments was <0.01% (v/v). Anti-PD-1 was purchased from BioXcell (West Lebanon, NH, USA).

### Generation of BM-Derived DCs

BALB/c BM-derived immature DCs (imDCs) were generated as described previously (21, 22, 36). Briefly, BM was harvested from the femurs and tibiae of mice and cultured in RPMI-1640 medium (Gibco-BRL) supplemented with 10% (v/v) FBS (Gibco-BRL) and 1% (w/v) PS in the presence of 20 ng/mL recombinant murine (rm) GM-CSF (R&D Systems, Minneapolis, MN, USA) and 10 ng/mL rmIL-4 (R&D Systems). On culture days 2 and 4, half of the medium was removed and replaced with fresh media containing cytokines. On day 6, imDCs were purified *via* positive selection with CD11c^+^-magnetic beads (Miltenyi Biotec, Auburn, CA, USA). Next, mature DCs were generated by further cultivation for 48 h of CD11c^+^ DCs with 10 ng/mL rm TNF-α (R&D Systems), 10 ng/mL rmIL-1β (R&D Systems), and 10 ng/mL rmGM-CSF (R&D Systems).

### Generation of Dying Myeloma Cell-Loaded DCs

The generation of dying myeloma cell-loaded DCs was performed as described previously (21, 22, 36). Briefly, MOPC-315 tumor cell death was induced by γ-irradiation (100 Gy; Gammacell-1000 Elite, MDS Nordion, Canada), followed by overnight culture in RPMI-1640 without FBS, and the cells were mixed with imDCs 2 h after maturation at a 2:1 ratio (DCs:dying tumor cells).

### Animal Vaccination

The following five vaccination groups were established: (1) PBS control, (2) DC vaccination, (3) DC vaccination plus anti-PD-1, (4) DC vaccination plus lenalidomide injection, and (5) DC vaccination plus anti-PD-1 and lenalidomide injection. On day 0, mice were injected subcutaneously in the right flank with 5 × 10^5^ MOPC-315 cells in a volume of 0.1 mL. After tumor growth, lenalidomide (0.5 mg/kg/day) was administrated orally once a day for 25 days with a 3-day break after the first 11-day dosing period. Each dose of DCs (1 × 10^6^/mouse) was injected subcutaneously into the left flank of BALB/c mice in a volume of 0.1 mL PBS on days 11, 15, 25, and 29; anti-PD-1 (250 μg/mouse) was injected intraperitoneally in a 0.1-mL volume on the same days as DC vaccination. To assess the antitumor status of vaccinated mice, we measured the length, width, and height of each tumor every 3 to 4 days using a Vernier caliper, and we calculated tumor volume using the standard formula for calculating the volume of an ellipsoid: *V* = 4/3π(length × width × height/8).

### Phenotypic Analysis of Splenocytes From Vaccinated Mice

At the indicated time points, mice were sacrificed and splenocyte phenotypes were characterized by their cell surface markers using fluorescently labeled monoclonal antibodies (mAbs) and analyzed by flow cytometry. Cells were stained with the following mAbs from eBioscience (San Diego, CA, USA): CD11b-FITC (clone: M1/70), CD11b-PE (clone: M1/70), Gr-1-PE (clone: RB6-8C5), CD4-APC (clone: RM4-5), CD4-PE (clone: H129.19), CD8-FITC (clone: 53-6.7), CD49b-PE (clone: Dx5), CD44-PE (clone: IM7), CD62L-FITC (clone: MEL-14), CD69-FITC (clone: H1.2F3), CD25-FITC (clone: CD25-4E3), Foxp3-APC (clone: MF23), F4/80-FITC (clone: BM8), CD274-PE (clone: MH5), and CD206-APC (clone: C068C2). Isotype-matched controls were run in parallel. Cell debris was eliminated by forward- and side-scatter gating. The samples were acquired on a FACSCalibur cell sorter (Becton Dickinson, Mountain View, CA, USA) and data were analyzed using WinMDI ver. 2.9 (Biology Software Net: http://en.bio-soft.net/other/WinMDI.html).

### Tumor Antigen-Specific CTL Activity of Vaccinated Mice

Tumor antigen-specific CTL activity was investigated as described previously (21, 22, 36). Briefly, splenocytes (1 × 10^6^) isolated from vaccinated mice 7 days after the final DC vaccination (day 36) were added to 24-well plates and restimulated with irradiated MOPC-315 cells (5 × 10^5^ cells) for 5 days in RPMI-1640 (Gibco-BRL) containing 10% FBS (Gibco-BRL) and 1% PS supplemented with 20 ng/mL rmIL-2 (R&D Systems). After restimulation, we assessed the splenocytes for tumor antigen-specific CTLs using a mouse IFN-γ enzyme-linked immunospot (ELISPOT) assay (BD Bioscience). The MOPC-315 cell line and NK-sensitive YAC-1 cell line were used as target cells.

### *In Vitro* Analysis of Cytokine Production in Vaccinated Mice

We determined cytokine (IFN-γ, IL-10, and TGF-β) production in vaccinated mice using the BD OptEIA™ enzyme-linked immunosorbent assay (ELISA; BD Bioscience). Supernatants from restimulated splenocytes of vaccinated mice and from single tumor cells of all vaccinated mice were assayed to measure the production of Th1- and Th2-polarizing cytokines. Each sample was analyzed in triplicate, and the mean absorbance for each set of standards and samples was calculated.

### Intracellular Staining Assay of Tregs and Macrophages Generated in the Spleens of Vaccinated Mice

To evaluate the proportion of Tregs and macrophages, 1 × 10^6^ splenocytes from vaccinated mice were harvested, washed, and stained with surface-staining antibodies of Tregs (CD4-PE and CD25-FITC) and macrophages (CD11b-FITC and F4/80-PE) for 30 min at 4°C. Fc block was added before incubation with surface-staining antibodies. Next, the cells were washed and permeabilized with Fixation/Permeabilization Solution 2 (eBioscience) for 30 min at room temperature. After washing twice, the cells were stained with an intracellular staining antibody, Tregs [Alexa Fluor-conjugated Foxp3 antibody (Miltenyi Biotec)] and macrophages (CD206-APC) for 30 min at room temperature. The samples were acquired on a FACSCalibur cell sorter (Becton Dickinson), and data were analyzed using WinMDI ver. 2.9.

### Statistical Analyses

We performed statistical analyses using GraphPad Prism 4 (La Jolla, CA, USA). *t*-Tests, one-way analysis of variance (ANOVA), and two-way ANOVA were used as appropriate. We analyzed the survival of vaccinated mice using SigmaPlot 10.0 (Systat Software, San Jose, CA, USA). *P* < 0.05 was considered significant. Values are expressed as means ± SDs.

## Results

### DC Vaccination in Combination With Lenalidomide and Anti-PD-1 Treatment Induced a Synergistic Anti-Myeloma Immunity Effect

**Figure 1:**
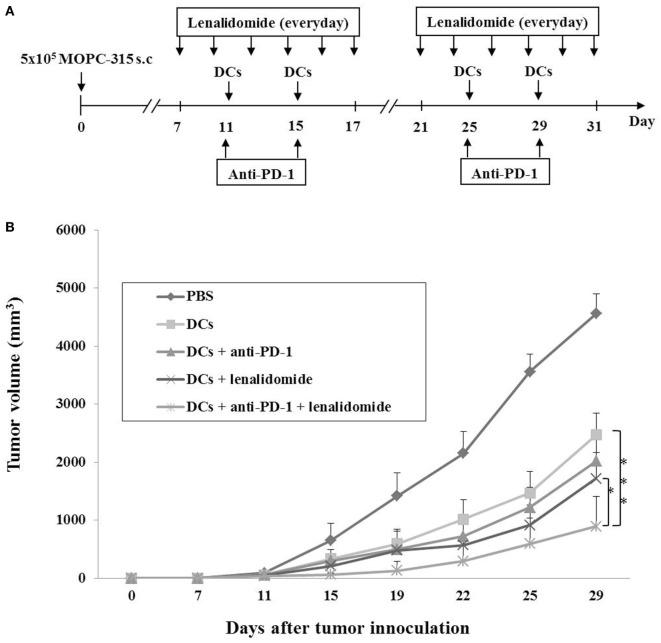
*In vivo* animal vaccination Five vaccination groups were established: (1) PBS control, (2) dying myeloma cell-loaded dendritic cell (DC) vaccination, (3) DC vaccination plus anti-PD-1, (4) DC vaccination plus lenalidomide, and (5) DC vaccination plus lenalidomide and anti-PD-1. On day 0, MOPC-315 cells (5 × 10^5^/mouse) were injected subcutaneously into the right flank of BALB/c mice. **(A)** Schematic representation of the combination of DCs plus lenalidomide and anti-PD-1. After tumor growth, lenalidomide (0.5 mg/kg/day) was administrated orally once a day for 25 days with a 3-day break after the first 11-day dosing period. Each dose of DCs (1 × 10^6^/mouse) was injected subcutaneously into the left flank of BALB/c mice in a volume of 0.1 mL PBS on days 11, 15, 25, and 29. Anti-PD-1 (250 μg/mouse) was injected intraperitoneally on the same days as DC vaccination. **(B)** Data are presented as mean ± SEM and are representative of two independent experiments. The combination of DCs plus lenalidomide and anti-PD-1 significantly inhibited tumor growth (*P < 0.05; ***P < 0.001 on day 29) and induced a long-term systemic anti-myeloma immune response (29 days).

Our previous study (36) demonstrated that DCs maturated with GM-CSF, TNF-α, and IL-1β expressed higher levels of several molecules related to DC maturation and produced higher levels of IL-12p70 and lower levels of IL-10 compared to imDCs. In this study, we established myeloma-bearing mice to evaluate the antitumor efficacy of DC-based immunotherapies. Before treatment, we observed that high levels of PD-L1 were expressed on MOPC-315 cell lines (Figure S1A in Supplementary Material). The established myeloma-bearing mice were initially treated with lenalidomide (0.25 or 0.5 mg/kg), PD-1 blockade (250 μg/mouse), and dying myeloma cell-loaded DCs as a single therapy (Figure 1A). All single treatment groups showed significant inhibition of tumor growth compared to the PBS control group (*P* < 0.05; Figures S1B,C in Supplementary Material). The combination therapy of DCs plus lenalidomide and PD-1 blockade was examined in an effort to more potently inhibit tumor growth in the murine myeloma model (Figure 1A). All tumor-bearing mice vaccinated with PBS showed rapid tumor growth that led to sacrifice within 3 weeks. By contrast, tumor-bearing mice vaccinated with DCs showed significantly inhibited tumor growth compared to the PBS control group. Treatment with the combination of DC vaccination plus lenalidomide and PD-1 blockade more strongly inhibited tumor growth (*P* < 0.05) compared to DCs, DCs + lenalidomide, DCs + PD-1 blockade, and lenalidomide + PD-1 blockade (Figure 1B; Figures S2A and S3A,B in Supplementary Material). Survival in mice that received the combination of DCs + lenalidomide + PD-1 blockade was significantly prolonged compared to that of mice received DCs, DCs + lenalidomide, DCs + PD-1 blockade, or lenalidomide + PD-1 blockade (Figures S2B and S3C in Supplementary Material). These results indicate that DCs + lenalidomide + PD-1 blockade induce a long-term systemic anti-myeloma immune response in the murine myeloma model.

### Activation of CTLs by DC Vaccination Plus Lenalidomide and PD-1 Blockade

**Figure 2:**
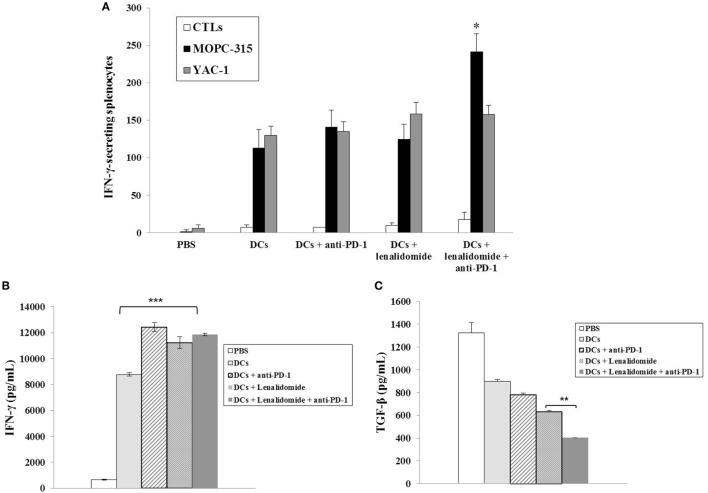
Activation of cytotoxic T lymphocytes (CTLs) and cytokine production induced by treatment with dendritic cells (DCs) plus lenalidomide and anti-PD-1. **(A)** The number of IFN-γ-secreting lymphocytes in the spleens of mice treated with PBS, DCs, DCs plus lenaldiomide, DCs plus anti-PD-1, and DCs plus lenalidomide and anti-PD-1 was counted using IFN-γ enzyme-linked immunospot assay. DC vaccination combined with lenalidomide and anti-PD-1 injection significantly increased the number of IFN-γ-secreting lymphocytes targeting MOPC-315 cells compared to the other groups (*P < 0.05). Cytotoxicity by natural killer (NK) cells, represented by the number of IFN-γ-secreting splenocytes against YAC-1 cells, was similar in all DC groups. These results indicate that the tumor inhibitory effects of DCs plus lenalidomide and anti-PD-1 resulted from the CTL-mediated response rather than the NK cell-mediated response. **(B)** IFN-γ and **(C)** TGF-β production in the splenocytes of vaccinated mice was evaluated by enzyme-linked immunosorbent assay. The combination of DCs plus lenalidomide and anti-PD-1 led to the production of higher levels of IFN-γ compared to PBS control and DC vaccination (***P < 0.001). By contrast, TGF-β production by DCs plus lenalidomide and anti-PD-1 was lower compared to the other groups (**P < 0.012). Data are shown as mean (pg/mL) ± SD of triplicate cultures from three independent experiments.

**Figure 3:**
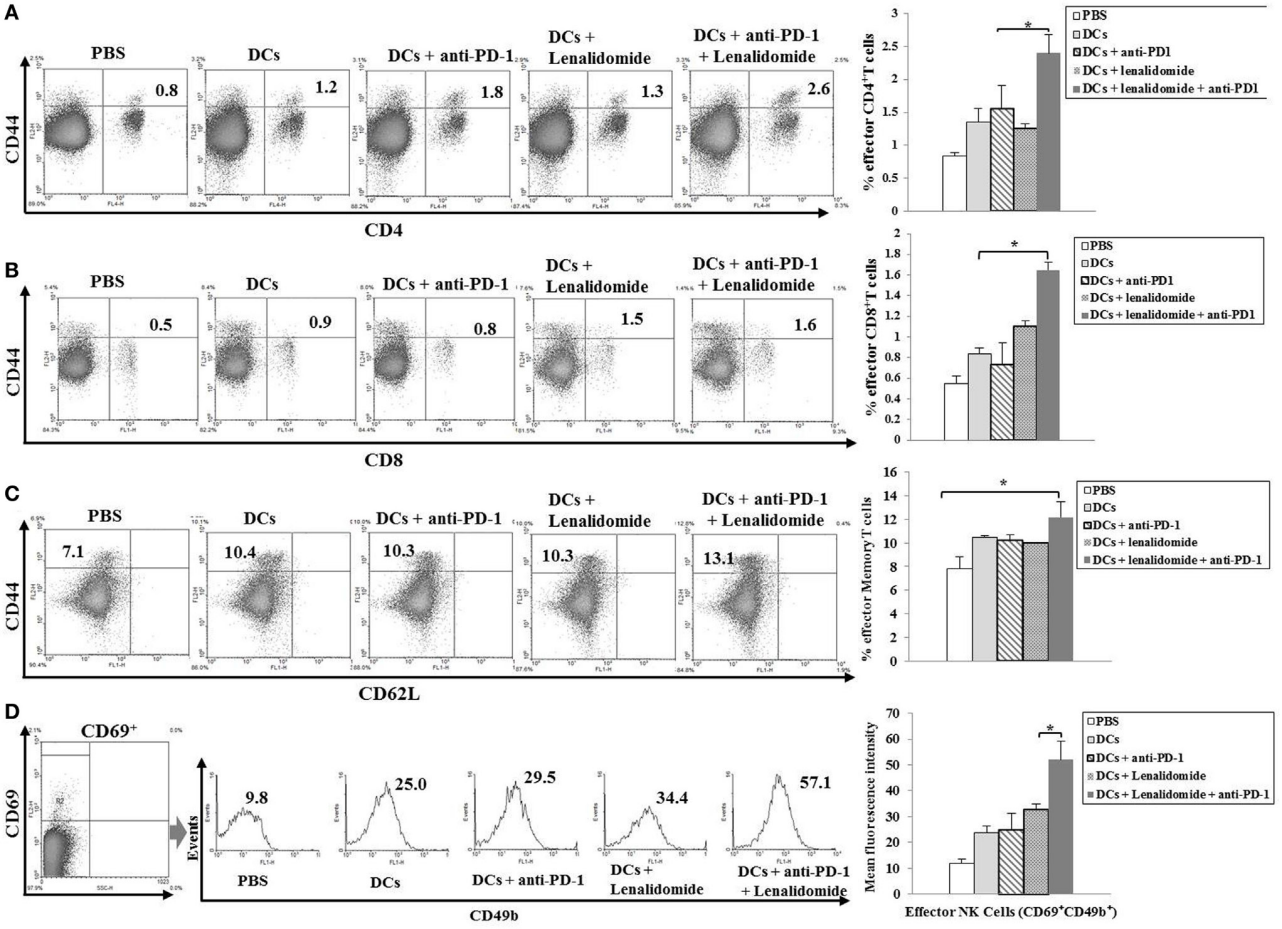
Induction of CD4^+^ T cells, CD8^+^ T cells, memory T cells, and natural killer (NK) cells in the spleens of mice treated with a combination of dendritic cells (DCs) plus lenalidomide and anti-PD-1. We measured proportions of **(A)** CD4^+^ T cells, **(B)** CD8^+^ T cells, **(C)** memory T cells, and **(D)** NK cells using flow cytometry (left panel) and compared them using quantitative bar graphs (right panel). The results revealed significant increases in effector cells in the DCs plus lenalidomide and anti-PD-1 combination group compared to the other groups (*P < 0.05). Data are representative of three independent experiments.

To investigate the CTL responses after DC vaccination, we prepared splenocytes and carried out IFN-γ ELISPOT assays. MOPC-315 and YAC-1 cells were used as target cells. Compared to the PBS control, DC vaccination, DCs + lenalidomide, DCs + PD-1 blockade, or DCs + lenalidomide + PD-1 blockade led to a significant increase in IFN-γ-secreting splenocytes against MOPC-315 and YAC-1 cells (*P* < 0.05). The combination of DCs + lenalidomide + PD-1 blockade showed the highest number of IFN-γ-secreting splenocytes against MOPC-315 cells compared to the PBS control, DCs, DCs + lenalidomide, DCs + PD-1 blockade, and lenalidomide + PD-1 blockade (*P* < 0.05; Figure 2A; Figure S4A in Supplementary Material). In addition, cytotoxicity by NK cells, represented by the number of IFN-γ-secreting splenocytes against YAC-1 cells, was similar in all groups that received DCs. These results indicate that the tumor inhibitory effects of DCs + lenalidomide + PD-1 blockade treatment resulted from CTL rather than NK responses. In this study, vaccination with DCs + lenalidomide + PD-1 blockade led to the production of higher levels of IFN-γ compared to the PBS control, DCs, or lenalidomide + PD-1 blockade group (Figure 2B; Figure S4B in Supplementary Material). By contrast, TGF-β production in the DCs + lenalidomide + PD-1 blockade group was significantly lower compared to that in the PBS control, DCs, DCs + lenalidomide, or DCs + PD-1 blockade group (Figure 2C; Figure S4C in Supplementary Material). These results suggest that the combination of DCs + lenalidomide + PD-1 blockade induced tumor-specific CTL responses enhances through Th1 polarization. Additionally, the DCs + lenalidomide + PD-1 blockade regimen significantly increased percentages of effector CD4^+^ T cells (Figure 3A; Figure S4D in Supplementary Material), effector CD8^+^ T cells (Figure 3B), effector memory T cells (Figure 3C; Figure S4E in Supplementary Material), effector NK cells (Figure 3D; Figure S4F in Supplementary Material), and M1 macrophages (Figure 5A; Figure S6A in Supplementary Material) compared to the other groups.

### Suppression of MDSCs, M2 Macrophages, and Regulatory T Cells (Tregs) by the Combination of DC Vaccination Plus Lenalidomide and PD-1 Blockade

**Figure 4:**
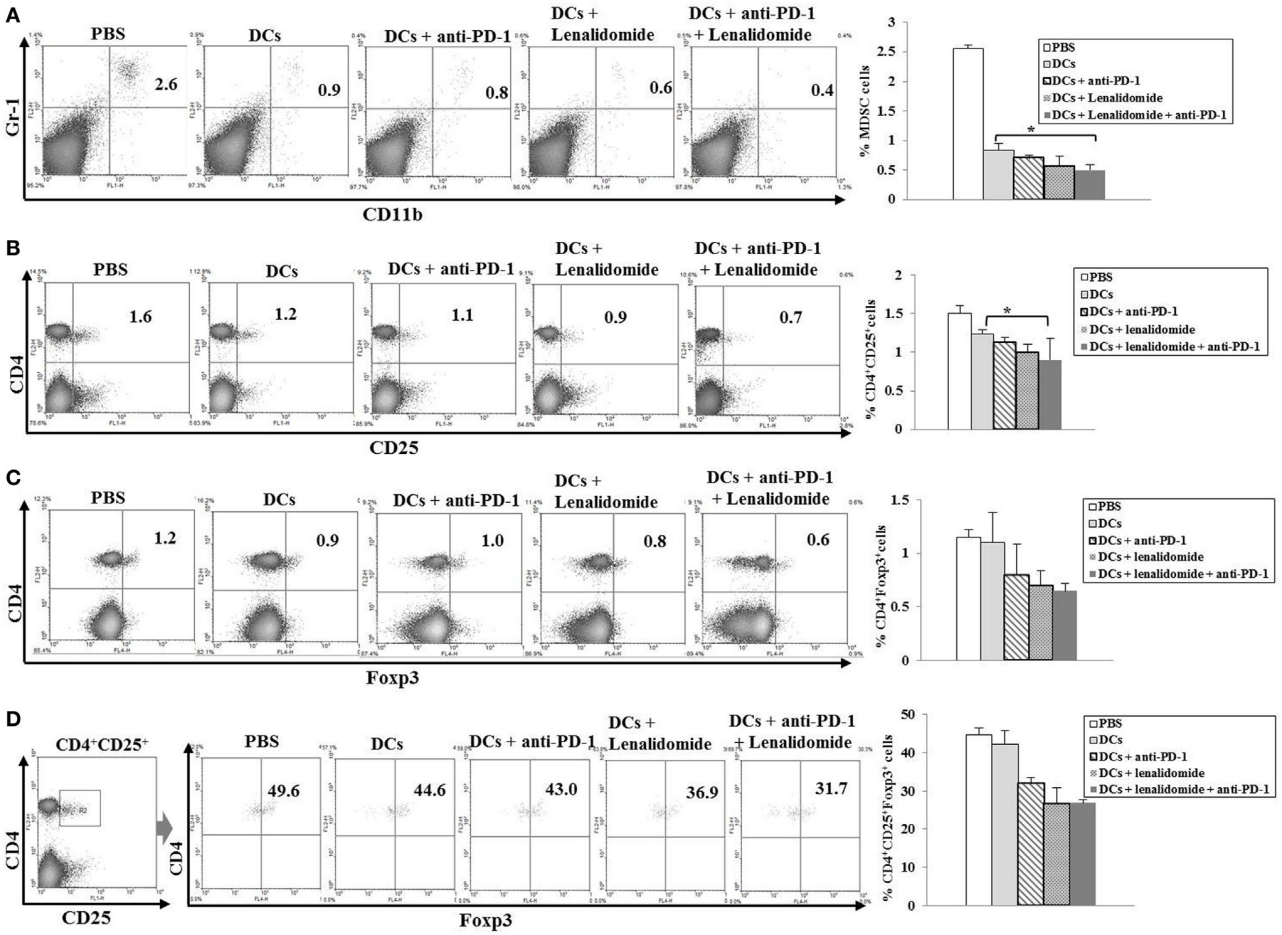
Inhibition of myeloid-derived suppressor cells (MDSCs) and regulatory T cells (Tregs) in the spleens of mice treated with dendritic cells (DCs) plus lenalidomide and anti-PD-1 We measured proportions of **(A)** MDSCs (CD11b^+^Gr-1^+^), **(B)** CD4^+^CD25^+^ Tregs, **(C)** CD4^+^Foxp3^+^ Tregs, and **(D)** CD4^+^CD25^+^Foxp3^+^ Tregs using flow cytometry (left panel) and compared them using quantitative bar graphs (right panel). The proportions of MDSCs and Tregs were significantly increased in the PBS control and DC vaccination groups compared to the groups injected with lenalidomide or anti-PD-1 after tumor inoculation. The DC vaccination plus lenalidomide and anti-PD-1 combination group showed significantly decreased proportions of splenic MDSCs and Tregs compared to the other groups (*P < 0.05). Data are representative of at least three experiments.

**Figure 5:**
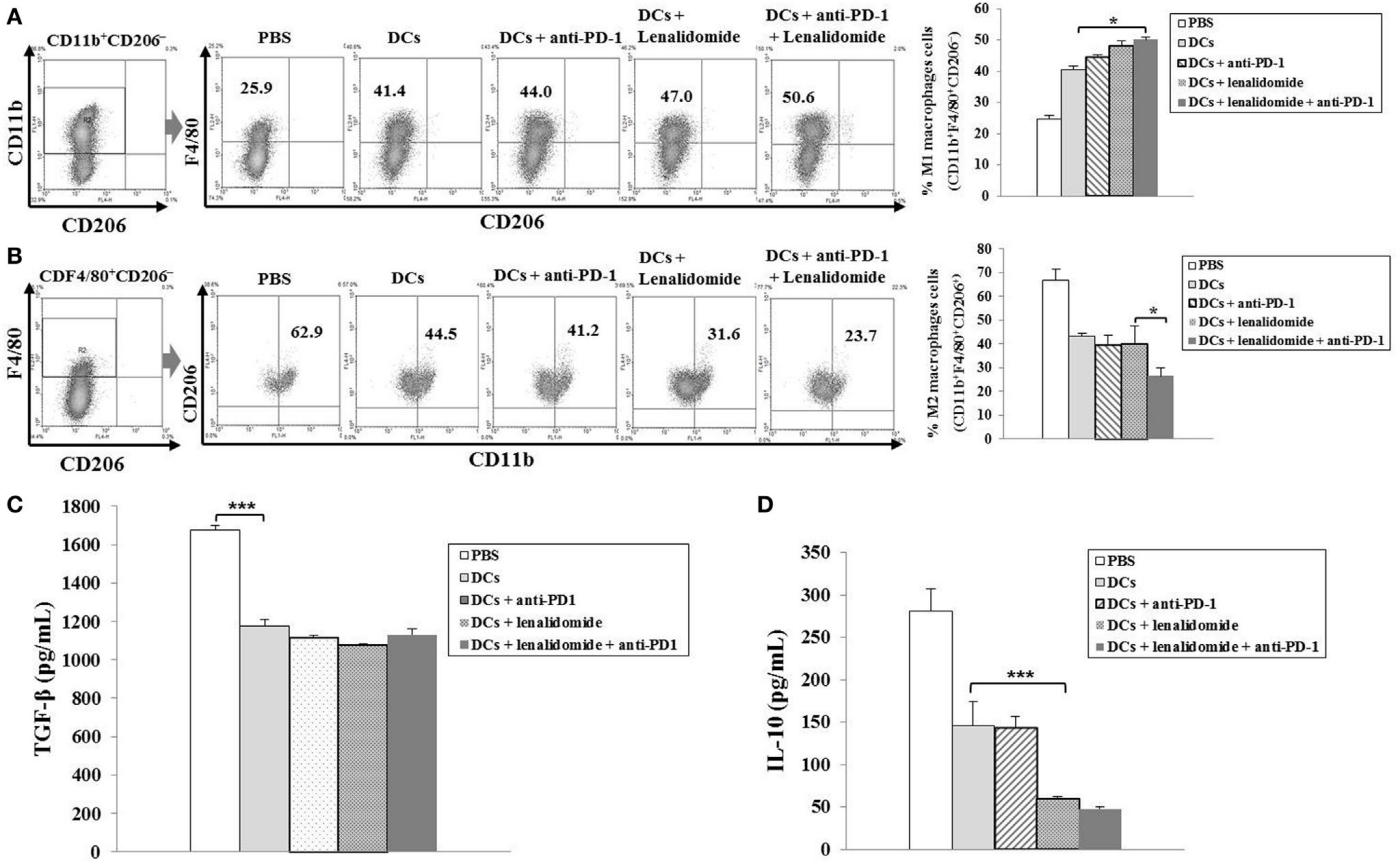
Enhanced M1 and impaired M2 macrophage polarization and reduced inhibitory cytokine production *via* vaccination with dendritic cells (DCs) plus lenalidomide and anti-PD-1 We measured proportions of **(A)** M1 macrophages (CD11b^+^F4/80^+^CD206^−^) and **(B)** M2 macrophages (CD11b^+^F4/80^+^CD206^+^) in the spleens of vaccinated tumor-bearing mice using flow cytometry (left panel) and compared them using quantitative bar graphs (right panel). The DCs plus lenalidomide and anti-PD-1 combination group exhibited the highest proportion of M1 macrophages and the lowest proportion of M2 macrophages compared to the other groups (*P < 0.05). Data are representative of three independent experiments. The production of **(C)** TGF-β and **(D)** IL-10 inhibitory cytokines in the tumors of tumor-bearing mice was evaluated by enzyme-linked immunosorbent assay. Compared to the treatments, PBS control led to the production of higher levels of TGF-β (***P < 0.001). However, the production of TGF-β did not differ significantly among the treatment groups. The production of IL-10 was significantly decreased in the DCs plus lenalidomide and anti-PD-1 combination therapy group compared to the other groups (***P < 0.001). Data are representative of at least three experiments.

To explore the immunological mechanisms underlying the enhanced tumor-specific immune response, we assessed the effects of combination therapy on the proportions of MDSCs (CD11b^+^Gr1^+^), M2 macrophages (CD11b^+^F4/80^+^CD206^+^ cells), and Tregs (CD4^+^CD25^+^FoxP3^+^ cells) in splenocytes. Percentages of MDSCs (Figure 4A; Figure S5A in Supplementary Material) and M2 macrophages (Figure 5B; Figure S6B in Supplementary Material) were dramatically reduced in all treatment groups compared to the PBS control group. The DCs + lenalidomide + PD-1 blockade group exhibited the lowest proportion of splenic MDSCs, and M2 macrophages (*P* < 0.05). The proportion of Tregs were significantly higher in the PBS control and DC vaccination groups compared to groups injected with lenalidomide + PD-1 blockade (*P* < 0.05). It is notable that the combination of DCs + lenalidomide + PD-1 blockade resulted in the lowest proportion of splenic Tregs (*P* < 0.05; Figures 4B–D; Figures S5B–D in Supplementary Material). These findings suggest that DCs + lenalidomide + PD-1 blockade enhances therapeutic antitumor immunity by also inhibiting immunosuppressive cells in the tumor microenvironment during the vaccination phases.

### Efficient Suppression of Inhibitory Cytokine Production by the Combination of DC Vaccination Plus Lenalidomide and PD-1 Blockade in the Tumor Microenvironment of Myeloma-Bearing Mice

To investigate the immunological mechanisms underlying the enhanced tumor-specific immune responses, we evaluated the inhibitory effects of the combination therapy (DC vaccination plus lenalidomide and PD-1 blockade) on the inhibitory cytokine production. Compared to the treatment groups, the PBS control group was significantly higher in the TGF-β production (*P* < 0.001). However, the production of TGF-β did not significantly differ among the treatment groups (Figure 5C; Figure S6C in Supplementary Material). In addition, the combination of DCs + lenalidomide + PD-1 blockade led to the production of the least IL-10 compared to other groups (*P* < 0.001; Figure 5D; Figure S6D in Supplementary Material), which suggests that the combination therapy of DC vaccination plus lenalidomide and PD-1 blockade changed the tumor microenvironment toward immunostimulatory by suppressing the production of inhibitory cytokines IL-10 and TGF-β.

## Discussion

Dendritic cell-based vaccines serve a promising immunotherapeutic weapon with the potential to prolong the survival of patients with incurable MM (2, 12). Several new tools have been developed and combined to improve clinical outcomes of DC vaccination against MM (17, 18). Recent studies have defined immune checkpoint PD-1/PD-L1 signaling as a key pathway regulating the critical balance between immune activation and tolerance (24, 37–40). The PD-1/PD-L1 pathway plays an important role in shaping the tumor-promoting, immunosuppressive microenvironment of MM. Rosenblatt et al. (41) reported that PD-L1 is highly expressed in plasma cells of MM patients but not in normal plasma cells. Our study confirmed that PD-L1 is also overexpressed on MOPC-315 cell lines (99%). Furthermore, significant PD-1 expression was observed in circulating T cells of advanced MM patients. Inhibition of the PD-1/PD-L1 signaling pathway induces an anti-MM immune response and can be a promising option for anti-myeloma therapy (42, 43).

The tumor microenvironment of MM promotes tumor cell growth and helps them escape from immune surveillance by actively suppressing anti-MM immune effector responses (1, 2). Lenalidomide, an immunomodulatory drug, inhibits the expression of PD-1 in NK cells, helper T cells, and CTLs of MM patients and downregulates the expression of PD-L1 in myeloma cell lines and primary myeloma cells (20, 34). Lenalidomide was shown to reduce PD-1 expression in all effector cells (CD4^+^ T cells, CD8^+^ T cells, NK cells, and NKT cells), and PD-L1 expression in MM cells, MDSC, and monocyte/macrophages in an *in vitro* experiment (35). Additionally, Patients undergoing treatment of lenalidomide demonstrated reduced PD-1 expression in CD8^+^ T cells (44). Moreover, lenalidomide plus PD-1/PD-L1 checkpoint blockade suppressed MDSCs and stroma-mediated MM growth and enhanced MM-specific cytotoxicity of immune effector cells in BM environments (35). Our previous studies demonstrated that DC-based vaccines were safe and induced the expansion of circulating CD4^+^ T cells and CD8^+^ T cells that are specific for tumor antigens, which was synergistically enhanced by the combination of lenalidomide (21, 22, 36). Our expectation was that the therapeutic efficacy of DC vaccination will be far more enhanced if both lenalidomide and PD-1 blockade are combined. Lenalidomide and anti-PD-1 antibody should synergistically improve the MM microenvironment, in which the host immune effector cells induced by the DC vaccination will exert anti-MM effects. This study, as expected, showed that DC vaccination combined to the lenalidomide and PD-1 blockade regiment further inhibited MM tumor growth, consequently prolonging the survival of tumor-bearing mice: the triple combination induced strong anti-myeloma CTL responses and increased the number of effector cells (CD4^+^ T cells, CD8^+^ T cells, NK cells, and M1 macrophages), while effectively discouraging suppressor cells (MDSCs, Tregs, and M2 macrophages) in the systemic immune compartment. These findings evidence the induction of systemic immune response potentially being able to eradicate disseminated diseases. DCs combined with lenalidomide and PD-1 blockade also heightened the anti-myeloma cell mediate immunity by inducing the Th1 polarization, as evidenced by the high-level production of IFN-γ, and by suppressing Th2 immune responses, as evidenced by the low-level production of IL-10 and TGF-β. Tregs, MDSCs, and M2 macrophages are major elements molding the potent immunosuppressive environment in tumor tissues. The inhibition of Treg, MDSC, and M2 macrophage accumulation in the spleen should further contribute to effective anti-myeloma cell mediate immunity in the systemic immune compartment by reciprocally activating DCs or CTLs.

Murine models of myeloma are critical tools to study the mechanisms of disease resistance, pathogenesis, and the development of new therapeutic strategies (45, 46). This study has some limitation to interpret data due to subcutaneous injection of MOPC-315 cells for making plasmacytoma rather than BM involvement model for myeloma.

In conclusion, this study suggests that lenalidomide plus PD-1 blockade treatment synergistically enhances the efficacy of DC vaccination in a murine myeloma model by inhibiting the generation of immunosuppressive cells and the Th2 immune response and enhancing effector cells and the Th1 immune responses. We hereby propose a framework for a more efficacious DC-based vaccination strategy against MM with the combination of immunomodulatory drug lenalidomide and anti-PD-1 antibody.

## Ethics Statement

All animal care, experiments, and euthanasia protocols were approved by the Chonnam National University Animal Research Committee.

## Author Contributions

M-CV, S-HJ, and J-JL designed the study. M-CV, T-HC, H-JL, TL, and H-SP performed the research and analyzed the data. M-CV, JR, and J-JL wore the article. M-CV, H-JK, and J-JL contributed intellectually to the research.

## Conflict of Interest Statement

The authors declare that the research was conducted in the absence of any commercial or financial relationships that could be construed as a potential conflict of interest.

## Supplementary Material

Figure S1 Antitumor efficacy of individual therapies in a model of murine myeloma. **(A)** We measured the levels of PD-L1 expressed on MOPC-315 cell lines using flow cytometry. MOPC-315 cell lines showed high-level expression of PD-L1 (99%). Representative histogram shows marker expression (shaded) compared with those of isotype control (black line). **(B)** Representative images of mice vaccinated with lenalidomide (0.25 or 0.5 mg/kg), anti-PD-1 (250 μg/mouse), and dying myeloma cell-loaded dendritic cells (DCs) as single treatments. **(C)** Data are shown as the mean ± SEM and are representative of two independent experiments. All single treatment groups showed significant inhibition of tumor growth compared to the PBS control (**P* < 0.05). Experiments consisted of five mice per group.

Figure S2 **(A)** Representative images of mice vaccinated with dendritic cells (DCs) plus lenalidomide and anti-PD-1 showed significant inhibition of tumor growth compared to the PBS control, DC vaccination, DCs plus anti-PD-1, and DCs plus lenalidomide groups. **(B)** The survival of the tumor-bearing mice is shown. The combination of DCs plus lenalidomide and anti-PD-1 significantly inhibited tumor growth (**P* < 0.05; ****P* < 0.001 on day 29) and induced a long-term systemic anti-myeloma immune response. Experiments consisted of five mice per group.

Figure S3 **(A)** Representative images of mice vaccinated with dendritic cells (DCs) plus lenalidomide and anti-PD-1 showed significant inhibition of tumor growth compared to mice treated with lenalidomide plus anti-PD-1. **(B)** Data are shown as the mean ± SEM and are representative of two independent experiments. **(C)** The survival of the tumor-bearing mice is shown. The combination of DCs plus lenalidomide and anti-PD-1 significantly inhibited tumor growth (**P* < 0.05 on day 25) and induced a long-term systemic anti-myeloma immune response. Experiments consisted of five mice per group.

Figure S4 Activation of cytotoxic T lymphocytes and natural killer (NK) cells, proportions of CD4^+^ T cells and memory T cells, NK cells, and cytokine production induced by a combination of dendritic cells (DCs) plus lenalidomide and anti-PD-1 **(A)** We counted the number of IFN-γ-secreting lymphocytes in the spleens of mice treated with lenalidomide plus anti-PD-1 and with DCs plus lenalidomide and anti-PD-1 using the IFN-γ enzyme-linked immunospot assay. The combination of DCs plus lenalidomide and anti-PD-1 significantly increased the number of IFN-γ-secreting lymphocytes targeting MOPC-315 and YAC-1 cells compared to treatment with lenalidomide plus anti-PD-1 (**P* < 0.05). **(B)** IFN-γ and **(C)** TGF-β production in the splenocytes of vaccinated mice was evaluated by enzyme-linked immunosorbent assay. The combination of DCs plus lenalidomide and anti-PD-1 led to the production of higher levels of IFN-γ compared to treatment with lenalidomide plus anti-PD-1 (****P* < 0.001). The production of TGF-β did not differ significantly between the two groups. Data are shown as the mean (pg/mL) ± SD of triplicate cultures from three independent experiments. We measured proportions of **(D)** CD4^+^ T cells, **(E)** memory T cells, and **(F)** NK cells using flow cytometry (left panel) and compared them using quantitative bar graphs (right panel). The results revealed significant increases in CD4^+^ T cells and memory T cells in the DCs plus lenalidomide and anti-PD-1 group compared to the lenalidomide plus anti-PD-1 group (**P* < 0.05; ****P* < 0.001). Percentages of NK cells did not differ significantly between the two groups. Data are representative of at least three experiments.

Figure S5 Inhibition of myeloid-derived suppressor cells (MDSCs) and Tregs in the spleens of mice treated with a combination of dendritic cells (DCs) plus lenalidomide and anti-PD-1. We measured proportions of **(A)** MDSCs (CD11b^+^Gr-1^+^), **(B)** CD4^+^CD25^+^ Tregs, **(C)** CD4^+^Foxp3^+^ Tregs, and **(D)** CD4^+^CD25^+^Foxp3^+^ Tregs using flow cytometry (left panel) and compared them using quantitative bar graphs (right panel). The DC vaccination plus lenalidomide and anti-PD-1 combination group showed decreased proportions of splenic MDSCs and Tregs compared to the lenalidomide plus anti-PD-1 group. Data are representative of at least three experiments.

Figure S6 Enhanced M1 and impaired M2 macrophage polarization and reduced inhibitory cytokine production by a combination of dendritic cells (DCs) plus lenalidomide and anti-PD-1. We measured proportions of **(A)** M1 macrophages (CD11b^+^F4/80^+^CD206^−^) and **(B)** M2 macrophages (CD11b^+^F4/80^+^CD206^+^) in the spleens of vaccinated tumor-bearing mice using flow cytometry (left panel) and compared them using quantitative bar graphs (right panel). The DCs plus lenalidomide and anti-PD-1 combination group showed significantly increased proportions of M1 macrophages compared to the lenalidomide plus anti-PD-1 group (**P* < 0.05). Percentages of M2 macrophages did not differ significantly between the two groups. Data are representative of at least three experiments. The production of **(C)** TGF-β and **(D)** IL-10 inhibitory cytokines in the tumors of tumor-bearing mice was evaluated by enzyme-linked immunosorbent assay. The production of IL-10 was significantly decreased in the DCs plus lenalidomide and anti-PD-1 combination therapy group compared to the lenalidomide plus anti-PD-1 group (****P* < 0.001). The production of TGF-β did not differ significantly between the two groups. Data are representative of at least three experiments.
