# Supplementary figures and images for: Lenalidomide and Programmed Death-1 Blockade Synergistically Enhances the Effects of Dendritic Cell Vaccination in a Model of Murine Myeloma
Source: Front Immunol. 2018 Jun 18;9:1370. doi: 10.3389/fimmu.2018.01370 (PMC6015916; doi:10.3389/fimmu.2018.01370)

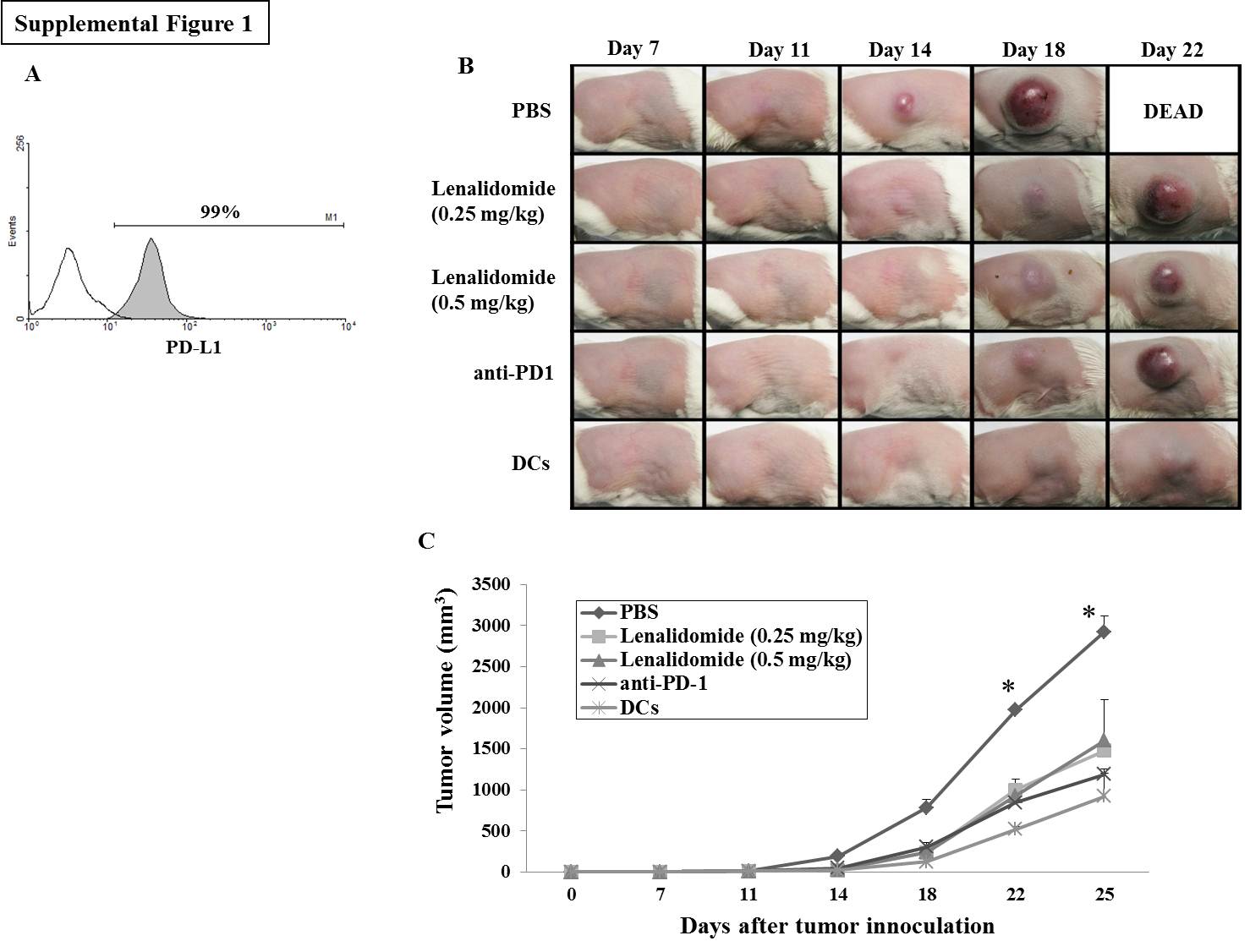

Supplement: Figure S1 — Antitumor efficacy of individual therapies in a model of murine myeloma. (A) We measured the levels of PD-L1 expressed on MOPC-315 cell lines using flow cytometry. MOPC-315 cell lines showed high-level expression of PD-L1 (99%). Representative histogram shows marker expression (shaded) compared with those of isotype control (black line). (B) Representative images of mice vaccinated with lenalidomide (0.25 or 0.5 mg/kg), anti-PD-1 (250 μg/mouse), and dying myeloma cell-loaded dendritic cells (DCs) as single treatments. (C) Data are shown as the mean ± SEM and are representative of two independent experiments. All single treatment groups showed significant inhibition of tumor growth compared to the PBS control (*P < 0.05). Experiments consisted of five mice per group. [file Image_1.jpeg]

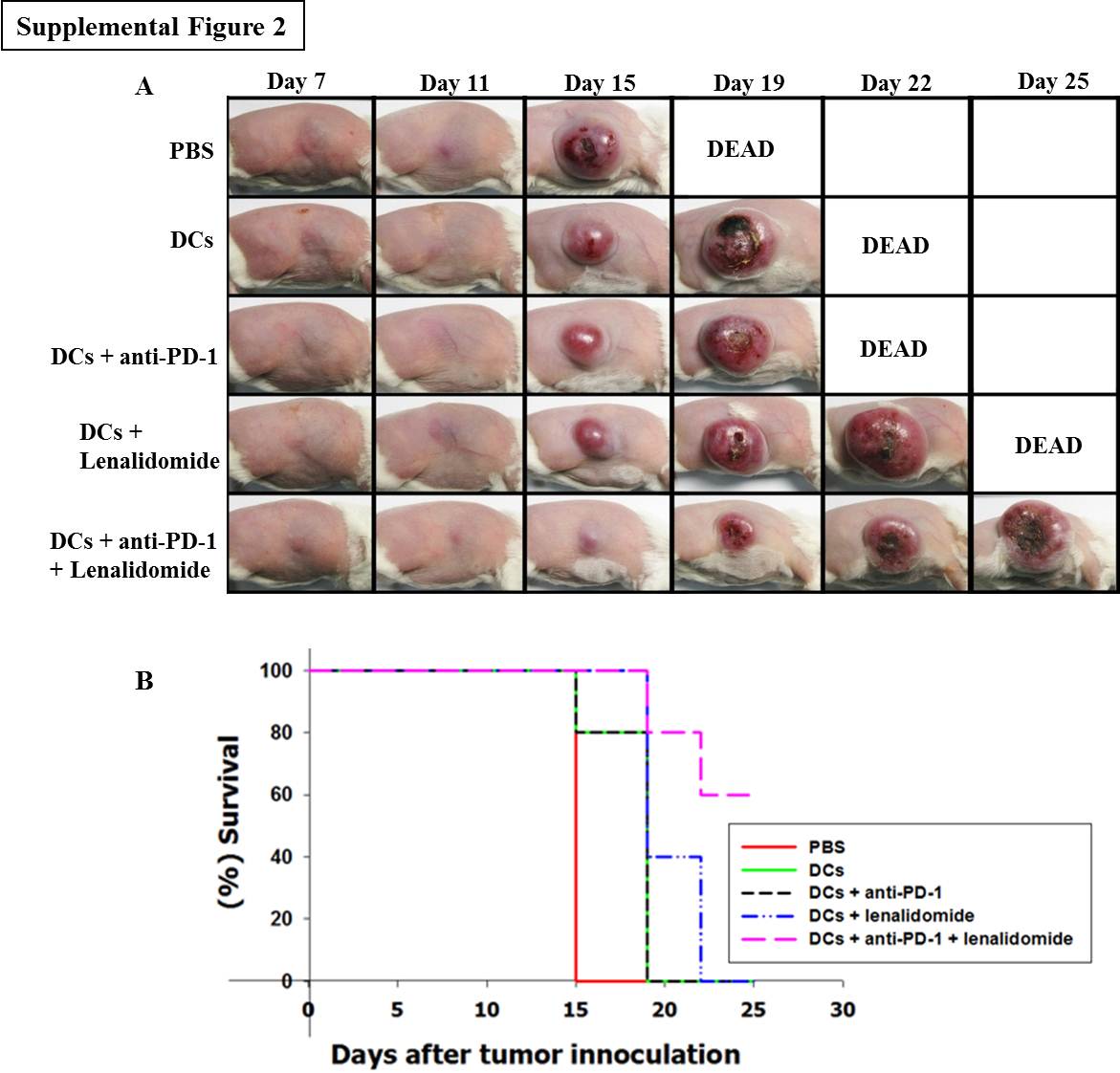

Supplement: Figure S2 — (A) Representative images of mice vaccinated with dendritic cells (DCs) plus lenalidomide and anti-PD-1 showed significant inhibition of tumor growth compared to the PBS control, DC vaccination, DCs plus anti-PD-1, and DCs plus lenalidomide groups. (B) The survival of the tumor-bearing mice is shown. The combination of DCs plus lenalidomide and anti-PD-1 significantly inhibited tumor growth (*P < 0.05; ***P < 0.001 on day 29) and induced a long-term systemic anti-myeloma immune response. Experiments consisted of five mice per group. [file Image_2.jpeg]

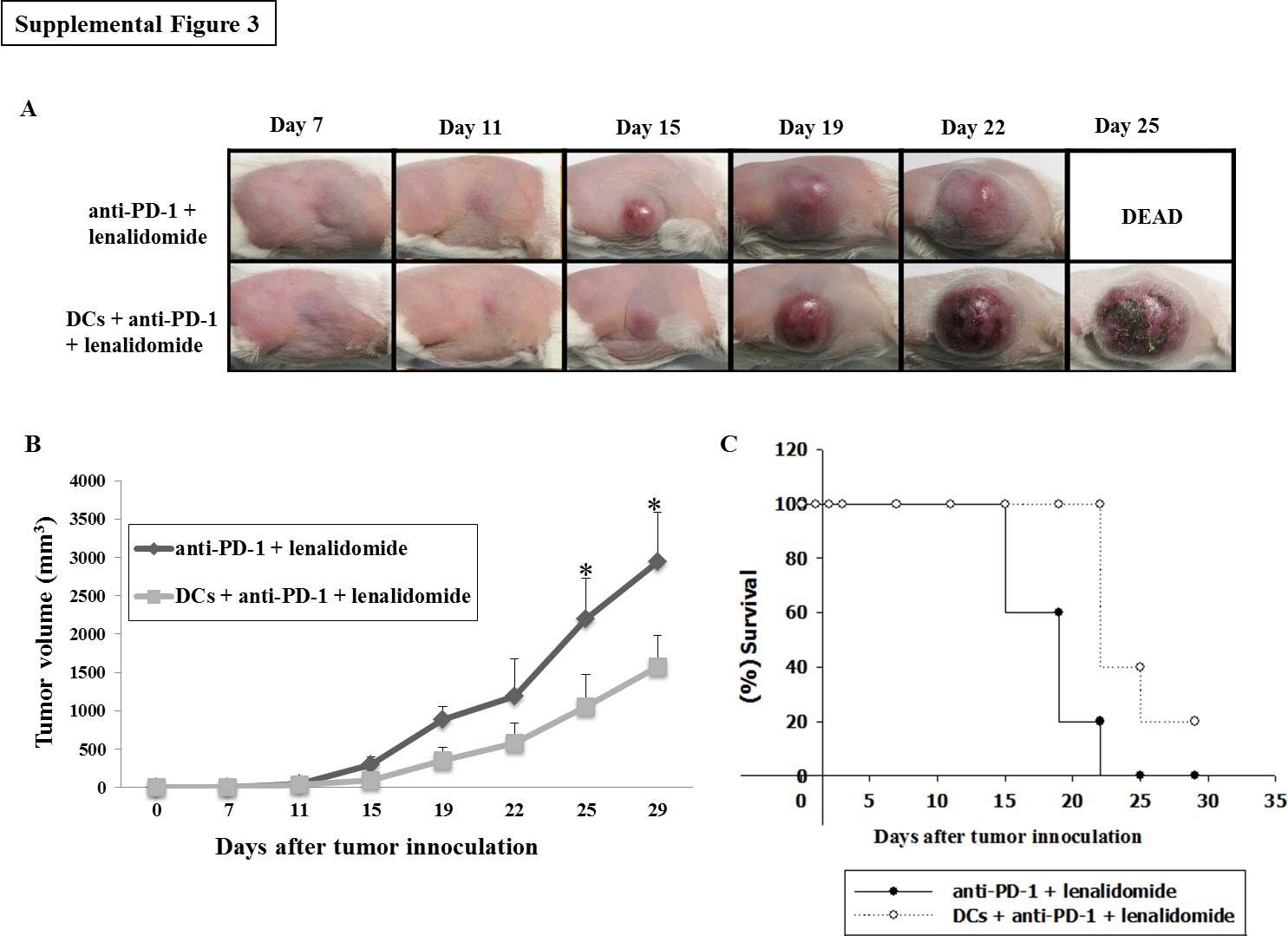

Supplement: Figure S3 — (A) Representative images of mice vaccinated with dendritic cells (DCs) plus lenalidomide and anti-PD-1 showed significant inhibition of tumor growth compared to mice treated with lenalidomide plus anti-PD-1. (B) Data are shown as the mean ± SEM and are representative of two independent experiments. (C) The survival of the tumor-bearing mice is shown. The combination of DCs plus lenalidomide and anti-PD-1 significantly inhibited tumor growth (*P < 0.05 on day 25) and induced a long-term systemic anti-myeloma immune response. Experiments consisted of five mice per group. [file Image_3.jpeg]

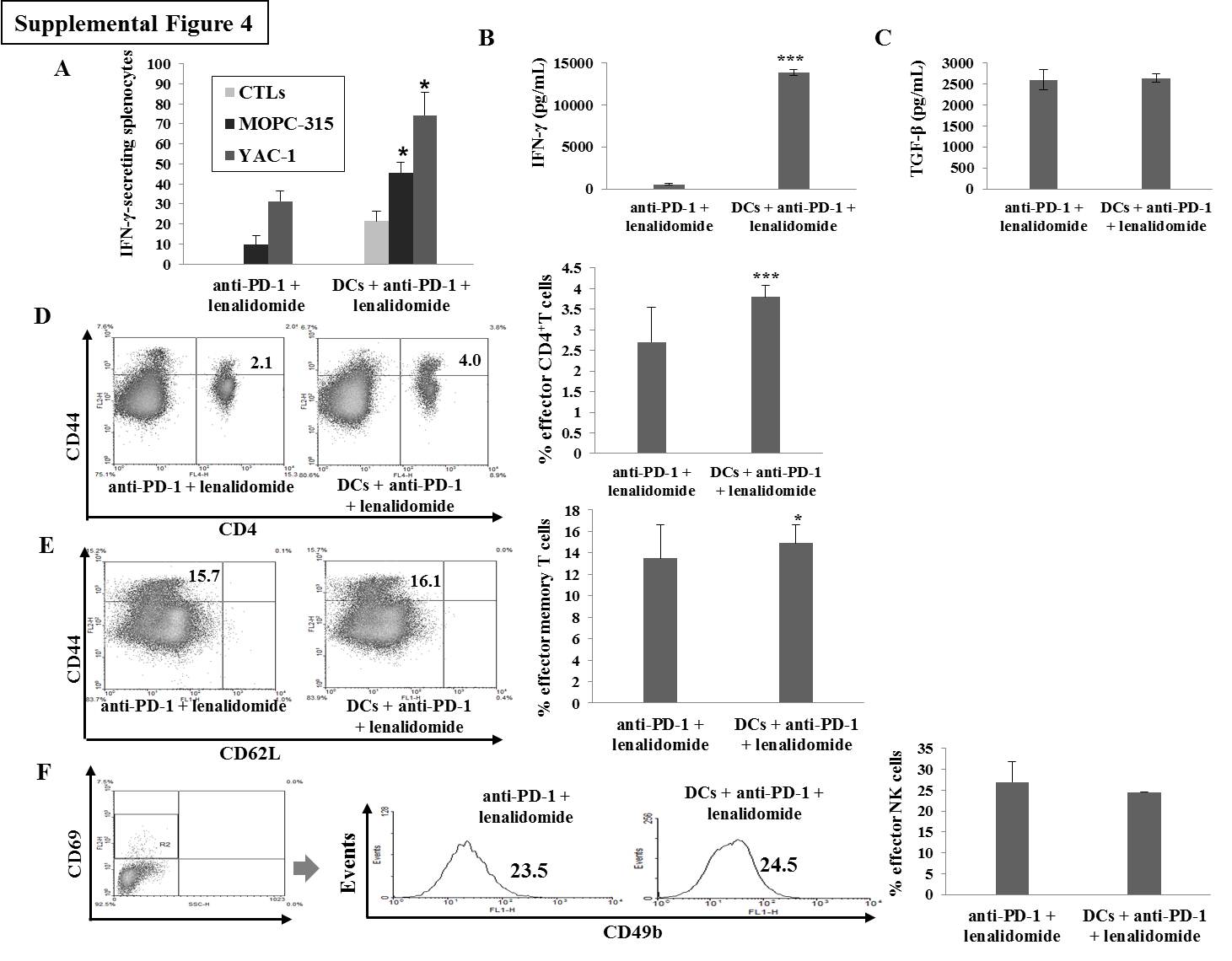

Supplement: Figure S4 — Activation of cytotoxic T lymphocytes and natural killer (NK) cells, proportions of CD4+ T cells and memory T cells, NK cells, and cytokine production induced by a combination of dendritic cells (DCs) plus lenalidomide and anti-PD-1 (A) We counted the number of IFN-γ-secreting lymphocytes in the spleens of mice treated with lenalidomide plus anti-PD-1 and with DCs plus lenalidomide and anti-PD-1 using the IFN-γ enzyme-linked immunospot assay. The combination of DCs plus lenalidomide and anti-PD-1 significantly increased the number of IFN-γ-secreting lymphocytes targeting MOPC-315 and YAC-1 cells compared to treatment with lenalidomide plus anti-PD-1 (*P < 0.05). (B) IFN-γ and (C) TGF-β production in the splenocytes of vaccinated mice was evaluated by enzyme-linked immunosorbent assay. The combination of DCs plus lenalidomide and anti-PD-1 led to the production of higher levels of IFN-γ compared to treatment with lenalidomide plus anti-PD-1 (***P < 0.001). The production of TGF-β did not differ significantly between the two groups. Data are shown as the mean (pg/mL) ± SD of triplicate cultures from three independent experiments. We measured proportions of (D) CD4+ T cells, (E) memory T cells, and (F) NK cells using flow cytometry (left panel) and compared them using quantitative bar graphs (right panel). The results revealed significant increases in CD4+ T cells and memory T cells in the DCs plus lenalidomide and anti-PD-1 group compared to the lenalidomide plus anti-PD-1 group (*P < 0.05; ***P < 0.001). Percentages of NK cells did not differ significantly between the two groups. Data are representative of at least three experiments. [file Image_4.jpeg]

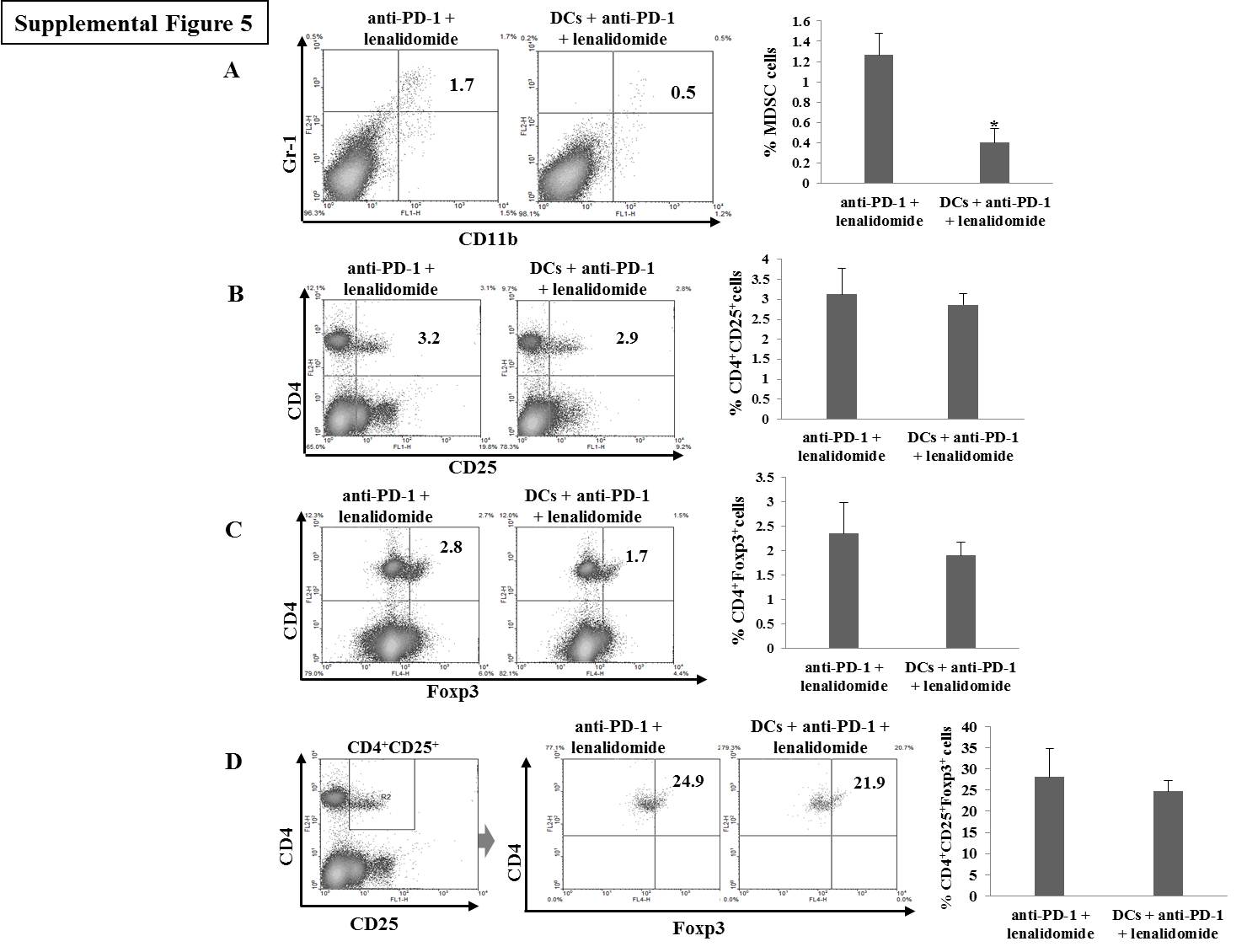

Supplement: Figure S5 — Inhibition of myeloid-derived suppressor cells (MDSCs) and Tregs in the spleens of mice treated with a combination of dendritic cells (DCs) plus lenalidomide and anti-PD-1. We measured proportions of (A) MDSCs (CD11b+Gr-1+), (B) CD4+CD25+ Tregs, (C) CD4+Foxp3+ Tregs, and (D) CD4+CD25+Foxp3+ Tregs using flow cytometry (left panel) and compared them using quantitative bar graphs (right panel). The DC vaccination plus lenalidomide and anti-PD-1 combination group showed decreased proportions of splenic MDSCs and Tregs compared to the lenalidomide plus anti-PD-1 group. Data are representative of at least three experiments. [file Image_5.jpeg]

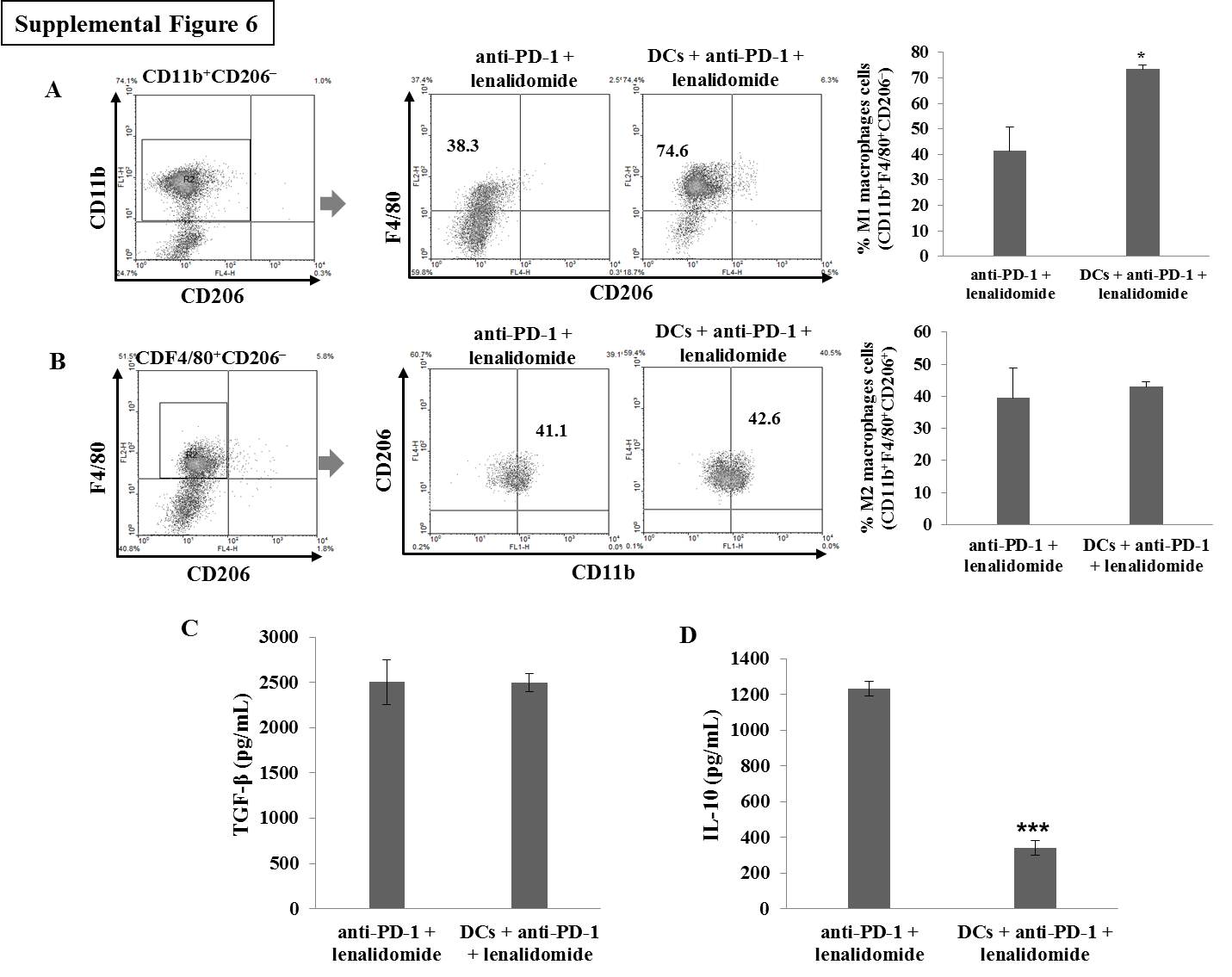

Supplement: Figure S6 — Enhanced M1 and impaired M2 macrophage polarization and reduced inhibitory cytokine production by a combination of dendritic cells (DCs) plus lenalidomide and anti-PD-1. We measured proportions of (A) M1 macrophages (CD11b+F4/80+CD206−) and (B) M2 macrophages (CD11b+F4/80+CD206+) in the spleens of vaccinated tumor-bearing mice using flow cytometry (left panel) and compared them using quantitative bar graphs (right panel). The DCs plus lenalidomide and anti-PD-1 combination group showed significantly increased proportions of M1 macrophages compared to the lenalidomide plus anti-PD-1 group (*P < 0.05). Percentages of M2 macrophages did not differ significantly between the two groups. Data are representative of at least three experiments. The production of (C) TGF-β and (D) IL-10 inhibitory cytokines in the tumors of tumor-bearing mice was evaluated by enzyme-linked immunosorbent assay. The production of IL-10 was significantly decreased in the DCs plus lenalidomide and anti-PD-1 combination therapy group compared to the lenalidomide plus anti-PD-1 group (***P < 0.001). The production of TGF-β did not differ significantly between the two groups. Data are representative of at least three experiments. [file Image_6.jpeg]
